# Host and viral determinants of feline immunodeficiency virus pathogenicity

**DOI:** 10.1186/1742-4690-10-S1-O39

**Published:** 2013-09-19

**Authors:** Brian J Willett, Pawel Bęczkowski, Martin Kraase, Nicola Logan, Elizabeth McMonagle, Annette L Litster, Margaret J Hosie

**Affiliations:** 1 Centre for Virus Research, University of Glasgow, Glasgow, UK; 2 Department of Veterinary Clinical Sciences, Purdue University, West Lafayette, IN 47907, USA

## Background

Infection with feline immunodeficiency virus (FIV) is mediated by attachment to CD134 (OX40) followed by a second interaction with CXCR4, the sole co-receptor for infection. However, the *in vivo* cell tropism of FIV expands with time post-infection, analogous to the shift in cell tropism observed with HIV-1 as co-receptor usage switches from CCR5 to CXCR4. Here, we ask whether alterations in the Env-CD134 interaction underpin the shift in FIV cell tropism and whether this is associated with disease progression.

## Results

Experimental transmission of a reconstituted quasispecies comprising viral variants with distinct modes of interaction with CD134 [1,2] revealed the selective expansion of variants bearing Envs typical of “early”, acute infection, binding CD134 through determinants in both cysteine rich domains (CRDs) 1 and 2. In contrast, variants with Envs typical of “late”, chronic infection (binding via CRD1 only) failed to thrive following experimental transmission. The basis for the defective replication of “late” variants did not lie in suppression by the humoral or cellular immune responses, but correlated with the nature of the virus-receptor interaction.

In order to assess whether our experimental observations on CD134 usage extended to natural infection of free-ranging cats, we characterized the receptor usage of viruses from two groups of cats naturally infected with FIV (n=44). Cats displaying clinical signs were more likely to harbour viral variants with a “late” phenotype (CRD1-dependent) than healthy cats. The emergence of CRD1-dependent variants coincided with declining health status, lower CD4 lymphocyte counts and led to shorter survival times. However, it was apparent that the shift from a CRD1&2 to CRD1-dependent interaction with CD134 was not a prerequisite for disease progression, as 25% of the cats that died during the study did not harbour Env variants displaying the “late” phenotype.

## Conclusions

The shift in CD134 usage observed following natural infection with FIV aligns with a model whereby transmitted viruses switch from CRD1&2-dependent binding to CD134, to a CRD1-dependent interaction as disease progresses. Whether this shift in receptor usage is a cause or consequence of disease progression remains to be established, however, receptor usage may serve as a prognostic indicator for disease progression.
